# Acquired focal choroidal excavation associated with multiple evanescent white dot syndrome: observations at onset and a pathogenic hypothesis

**DOI:** 10.1186/1471-2415-14-135

**Published:** 2014-11-20

**Authors:** Yuki Hashimoto, Wataru Saito, Kousuke Noda, Susumu Ishida

**Affiliations:** Department of Ophthalmology, Hokkaido University Graduate School of Medicine, Sapporo, Japan; Department of Ocular Circulation and Metabolism, Hokkaido University Graduate School of Medicine, Nishi 7, Kita 15, Sapporo, Kita-ku, 060-8638 Japan

**Keywords:** Choroidal thickness, Enhanced depth imaging optical coherence tomography, Focal choroidal excavation, Multiple evanescent white dot syndrome

## Abstract

**Background:**

The mechanism underlying focal choroidal excavation (FCE) remains largely unknown. We evaluated the sequential progression of FCE generation using enhanced depth imaging optical coherence tomography (EDI-OCT) in a patient with multiple evanescent white dot syndrome (MEWDS).

**Case presentation:**

A 37-year-old woman suffered MEWDS in the right eye. EDI-OCT showed the loss of photoreceptor inner segment/outer segment junction line, detachment between the retinal pigment epithelium (RPE) and Bruch’s membrane, and dome-shaped, moderately reflective, focal photoreceptor-layer lesions corresponding to perifoveal white dots. The region with pigment epithelium detachment involved RPE/Bruch’s membrane ruptures. After 1 month, almost all white dots spontaneously resolved together with improvements of the perifoveal OCT findings. Interestingly, perifoveal region developed a conforming-type FCE. An abnormal hyper-reflective lesion on OCT, regarded as fibrosis formation, simultaneously appeared within the choroid below the FCE and subsequently increased in size.

**Conclusions:**

These results suggest that the RPE/Bruch’s membrane disruption due to chorioretinal abnormalities and subsequent intrachoroidal scar formation play a role in the pathogenesis on an acquired FCE.

## Background

Multiple evanescent white dot syndrome (MEWDS) is a chorioretinal disease characterized by multiple subretinal, small, white dots extending from the posterior pole to the midperiphery [1]. Outer retinal morphology is also transiently impaired [2]. Moreover, multiple hypofluorescent spots on indocyanine green angiography (ICGA) [3] and increased choroidal thickness on enhanced depth imaging optical coherence tomography (EDI-OCT) were observed in the acute phase of MEWDS, suggesting the involvement of inflammation at the outer retina and choriocapillaris [4].

Focal choroidal excavation (FCE) is an excavation of the choroid, which can be detected with OCT [5, 6]. It is divided into the conforming and non-conforming types, depending on whether there is a separation between the neural retina and the retinal pigment epithelium (RPE) [6]. FCE is associated with various diseases, including age-related macular degeneration and central serous chorioretinopathy [5–8]. However, the mechanism underlying FCE formation remains largely unknown, including whether FCE are congenital or acquired [6]. In the present study, we present a case of MEWDS-associated acquired FCE, in which the development of an FCE was observed on EDI-OCT.

## Case presentation

**Figure 1:**
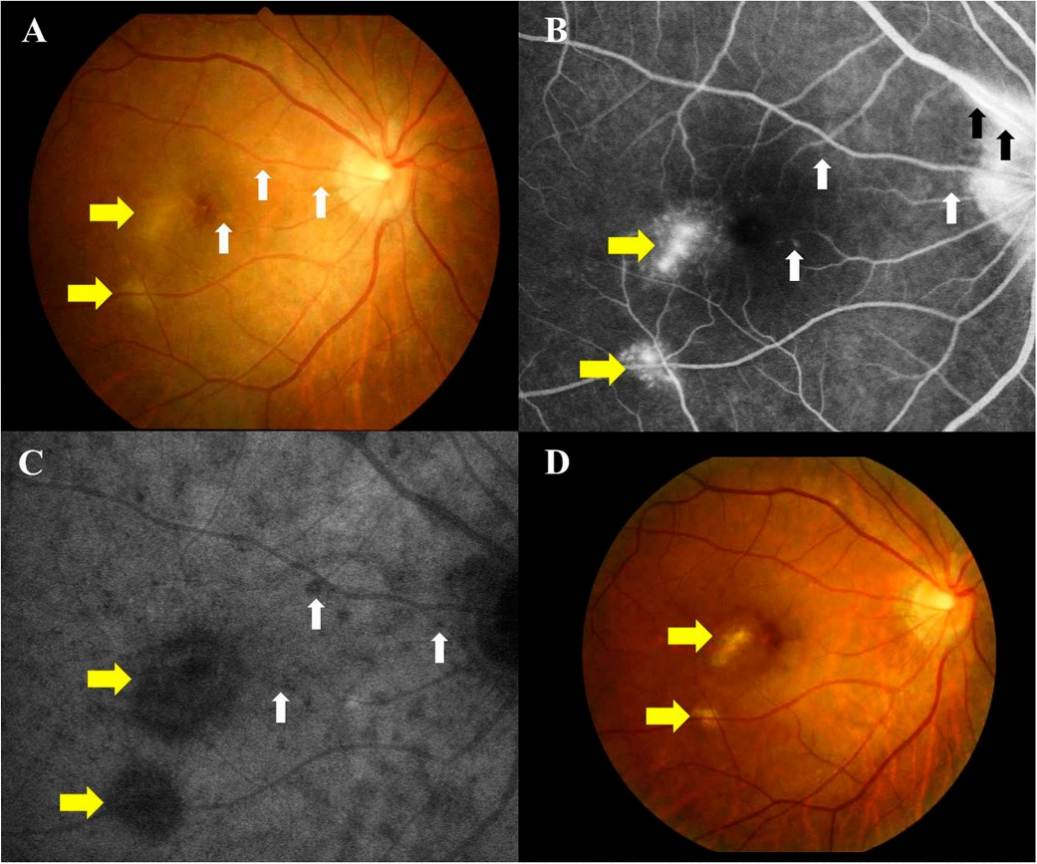
**Photographs of the right eye in a patient with multiple evanescent white dot syndrome at the initial visit (A-C) and 3 months later (D). A**, Multiple white dots (white arrows) with foveal granularity extending from the posterior pole to the midperiphery were seen. Relatively large white dots were also observed in the temporal fovea (yellow arrows). **B**, Late-phase fluorescein angiography showing hyperfluorescence corresponding to white dots (white and yellow arrows), retinal vasculitis (black arrows), and optic disc staining. **C**, Late–phase indocyanine green angiography images showing multiple hypofluorescent spots scattered over a wider area than the white dots (white and yellow arrows). **D**, White dots spontaneously disappeared, but large white dots (yellow arrows) in the temporal fovea developed scars.

**Figure 2:**
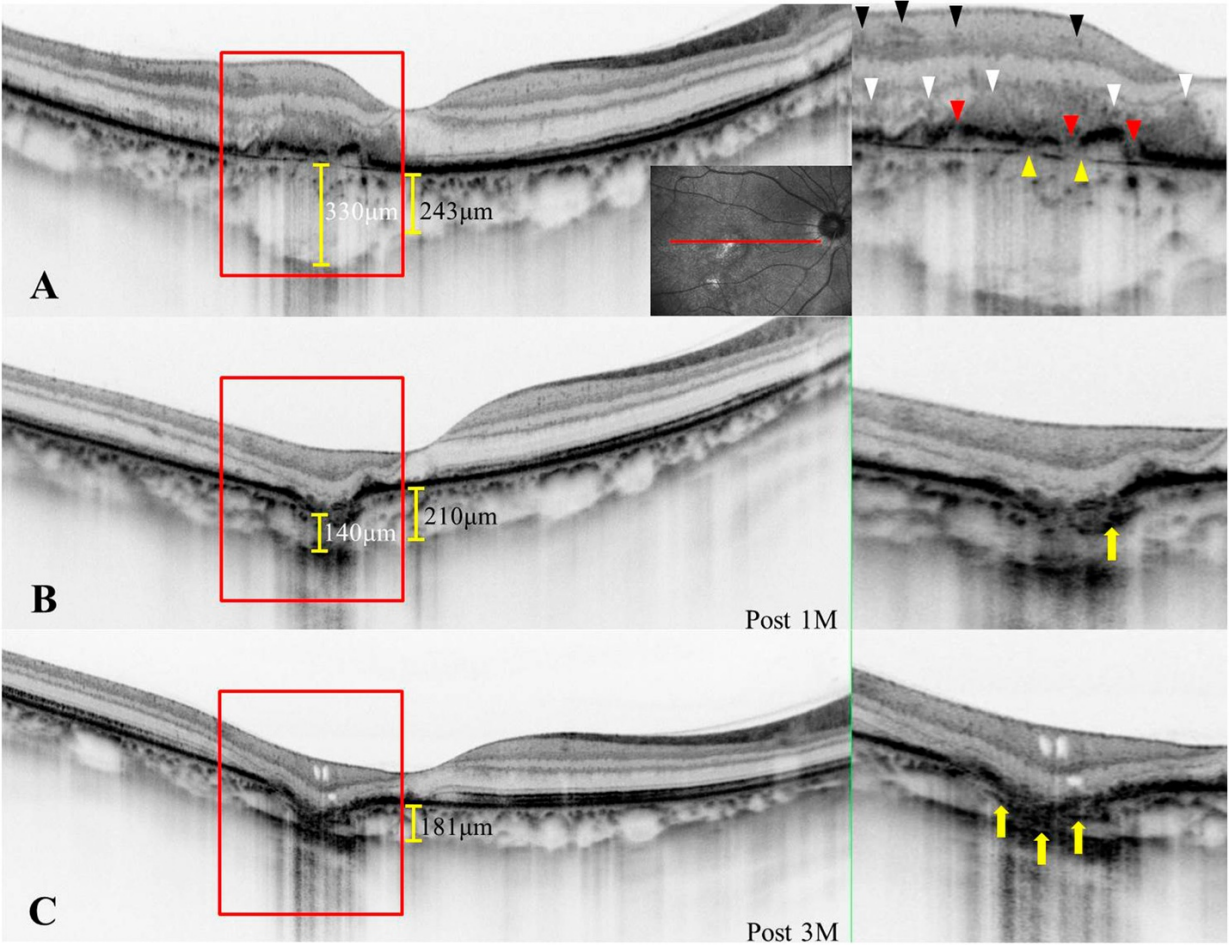
**Horizontal enhanced depth imaging optical coherence tomography (EDI-OCT) images across the fovea of the right eye.** Right panels are magnified views of the area indicated in the left panels. **A**, At the initial visit, ganglion cell layer (GCL) hyper-reflectivity (black arrowheads), photoreceptor inner segment/outer segment junction boundary loss, detachment between the retinal pigment epithelium (RPE) and Bruch’s membrane, moderately reflective, dome-shaped focal lesions (white arrowheads) in the photoreceptor layer were present and corresponded to the large white dot at the temporal fovea. The site with pigment epithelium detachment involved the RPE/Bruch’s membrane ruptures (red and yellow arrowheads). Choroidal thickness at the fovea and the adjacent temporal lesion was 243 and 330 μm, respectively. **B**, One month later, the pigment epithelium detachment and abnormal GCL hyper-reflectivity spontaneously resolved. Focal lesions in the photoreceptor layer also markedly decreased. However, this region developed a conforming-type focal choroidal excavation (FCE) and the choroidal thickness at the fovea and the FCE lesion decreased to 210 and 140 μm, respectively. An abnormal hyper-reflective lesion also appeared within the choroid beneath the FCE (arrow). **C**, Three months after the initial visit, focal lesions in the photoreceptor layer almost resolved, but cystoid changes in the inner retinal layers and marked outer layer thinning occurred. The abnormal hyper-reflective lesion (yellow arrows) in the region of the scar within the choroid increased in size compared to that at 1 month (Figure 2B, arrow). The subfoveal choroidal thickness further decreased to 181 μm.

**Figure 3:**
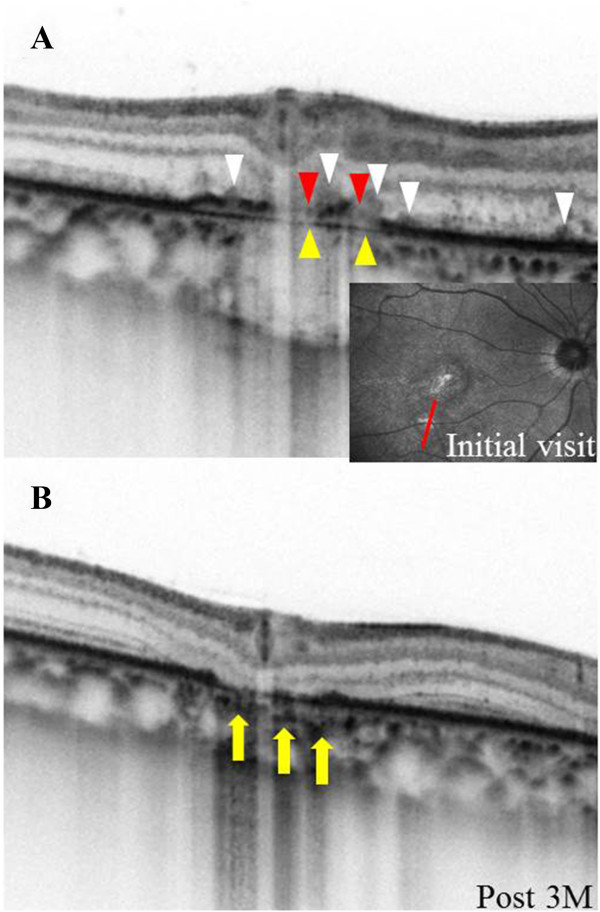
**EDI-OCT images through a white dot at the inferotemporal side of the fovea. A**, At the initial visit, the white dot involved inner and outer retinal morphological abnormalities including pigment epithelium detachment and moderately reflective focal lesions (white arrowheads) within photoreceptor layer as well as the other white dot at the temporal fovea (Figure 2A). However, there was no apparent rupture of Bruch’s membrane (yellow arrowheads) underlying the damaged RPE (red arrowheads). **B**, Three months later, the lesion showed the resolution of the pigment epithelium detachment and the appearance of an abnormal hyper-reflective lesion within the choroid (arrows), but no apparent sign of FCE development.

A 37-year-old woman with moderate myopia presented with complaints of blurred vision and photopsia in her right eye for 1 week. She had no antecedent flu-like illness. The patient’s medical and family histories were unremarkable. Best-corrected visual acuity (BCVA) with a Japanese standard Landolt visual acuity chart was 0.09 OD and 1.2 OS. Left eye showed normal appearance. Slit lamp examination showed 2+ cells in the anterior vitreous OD. Funduscopic examination showed foveal granularity and multiple white dots (Figure 1A, white arrows) extending from the posterior pole to the retinal midperiphery. Some white dots in the temporal fovea were relatively large (Figure 1A, yellow arrows). Fluorescein angiography (FA) showed hyperfluorescent spots (Figure 1B, arrows) from the initial phase, corresponding to the white dots, and the staining of retinal veins and the optic disc in the late phase. ICGA was normal in the early phase, but in the late phase numerous hypofluorescent spots were scattered over a wider area with and without white dots (Figure 1C). Goldmann perimetry showed a blind spot enlargement and a small central scotoma OD. Additionally, EDI-OCT showed hyper-reflective lesions in the ganglion cell layer (GCL) (Figure 2A, black arrowheads), the loss of photoreceptor inner segment/outer segment junction (IS/OS) line, detachment between the RPE and Bruch’s membrane, moderately reflective, dome-shaped focal lesions within the photoreceptor layer (Figures 2A and 3A; white arrowheads), and locally thickened choroid compared with neighboring areas, corresponding to the large white dots in the temporal fovea and inferotemporal perifovea with pigment epithelium detachment. Multiple ruptures of the RPE and Bruch’s membrane were apparently observed in the temporal lesion (Figure 2A, red and yellow arrowheads), whereas Bruch’s membrane underlying the damaged RPE was found to be continuous in the inferotemporal lesion (Figure 3A, red and yellow arrowheads). Choroidal thickness at the fovea and the adjacent temporal lesion was 243 and 330 μm OD, respectively (Figure 2A). The patient received a diagnosis of MEWDS OD.

The white dots spontaneously resolved 1 month after the initial visit, with visual acuity improving to 0.2 OD. The pigment epithelium detachment and abnormal GCL hyper-reflective lesions completely resolved and the photoreceptor-layer focal lesions markedly decreased in the temporal fovea (Figure 2B). Interestingly, a conforming FCE, without an intervening hyporeflective space between the photoreceptor tips and the RPE [4], developed in the same region (Figure 2B). Choroidal thickness at the fovea and the FCE lesion decreased to 210 and 140 μm, respectively (Figure 2B). An abnormal hyper-reflective lesion also appeared in the choroid below the FCE (Figure 2B, arrow) and increased in size 2 months after the initial visit.

Three months after the initial visit, BCVA improved to 0.4 and scars formed in the temporal and inferotemporal lesions (Figure 1D, yellow arrows). On ICGA, almost all hypofluorescent lesions disappeared, but the scarred sites exhibited hypofluorescent spots from the initial phase. On EDI-OCT images, the focal lesions in the photoreceptor layer almost resolved (Figures 2C and 3B), but cystoid changes emerged in inner retinal layers together with marked thinning of outer retinal layers (Figure 2C). The choroid below the temporal and inferotemporal scars developed massive hyper-reflective lesions (Figures 2C and 3B; yellow arrows), and the subfoveal choroidal thickness further decreased to 181 μm (Figure 2C). The inferotemporal perifoveal lesion did not develop any apparent sign of FCE (Figure 3B), in contrast to the temporal foveal lesion (Figure 2B,C). Twelve months after the initial visit, BCVA was 0.7 OD and the disrupted IS/OS line at the fovea partially improved. No recurrence of white dots or choroidal neovascularization had ever been observed.

## Discussion

MEWDS is a disease attributable to inflammation at the outer retina and choriocapillaris with features of transient impaired outer retinal morphology, multiple hypofluorescent spots on ICGA, and increased choroidal thickness on EDI-OCT in the acute phase [3, 4]. To the best of our knowledge, this is the first report of FCE development secondary to MEWDS. Moreover, we evaluated in detail the sequential progression of a conforming-type FCE on EDI-OCT images.

Margolis et al. hypothesized that FCEs may be congenital posterior-segment malformations because of an intact IS/OS and a scar-free choriocapillaris [6]. However, in the current case, outer retinal and inner choroidal layers were affected, the RPE and Bruch’s membrane were disrupted, and choroidal thickness increased prior to FCE development in the temporal perifovea. A choroidal hyper-reflective lesion appeared concurrently with FCE formation and subsequently increased in size, suggesting the presence of fibrous tissues within the choroid, likely due to resolved inflammation. These results suggest that active inflammation had spread from the outer retina to the inner choroid in this region. Bruch’s membrane is the basement membrane of both the RPE and the choriocapillaris, which contains supporting elastic and collagen fibers [9]. Taken together, we hypothesized that the following pathogenic events triggered FCE formation in the present case. First, high levels of inflammation occurred at the outer retina and the inner choroid, causing subsequent impairment of the RPE and Bruch’s membrane. Second, outer retinal and choroidal tissues adhered to each other through the RPE/Bruch’s membrane ruptures. Third, intraocular pressure resulted in photoreceptor protrusion through these holes into the choroid. Simultaneously, contraction of fibrotic lesions, which had stemmed from inflammation within the choroid, drew the overlying retinal tissues backward. These sequential progression may have allowed retinal tissue herniation towards the choroid to cause a conforming FCE. On the other hand, when a non-conforming FCE develops, the absence of adhesion between the neural retina and the RPE may keep photoreceptor tips in place. Further studies on various FCE-causing diseases are needed to elucidate the pathogenic process of FCEs because underlying etiologies may vary according to background diseases.

## Conclusions

We documented a patient with MEWDS who developed an acquired FCE during the follow-up period. Images obtained with EDI-OCT suggest that the RPE and Bruch’s membrane impairment, following chorioretinal inflammation, may play a role in the pathogenesis of FCEs.

## Consent

Written informed consent was obtained from the patient for publication of this Case report and any accompanying images. A copy of the written consent is available for review by the Editor of this journal.

## Abbreviations

MEWDS: Multiple evanescent white dot syndrome
ICGA: Indocyanine green angiography
EDI-OCT: Enhanced depth imaging optical coherence tomography
FCE: Focal choroidal excavation
RPE: Retinal pigment epithelium
BCVA: Best-corrected visual acuity
FA: Fluorescein angiography
GCL: Ganglion cell layer
IS/OS: Photoreceptor inner/outer segment junction
